# Letter to the Editor: Separating LLM repeatability from expert concordance in radiomics quality assessment

**DOI:** 10.1186/s41747-026-00858-0

**Published:** 2026-11-13

**Authors:** Bo Yang, Lingbo Meng, Jiageng Shen

**Affiliations:** 1 Department of Radiology, First Affiliated Hospital of Dalian Medical University, Dalian, China; 2 Department of Urology, First Affiliated Hospital of Dalian Medical University, Dalian, China

Dear Editor,

We read with interest the study by Garello et al which assessed the methodological quality of cardiac CT and MRI radiomics studies using the METhodological RadiomICs Score (METRICS) and the radiomics quality score (RQS) and compared expert assessments with ChatGPT 5.1 Thinking [1]. The comparison of complementary appraisal instruments is valuable and identifies clinically relevant discordance between human and large language model (LLM) assessments. Before these findings are interpreted as support for scalable LLM-assisted appraisal, an additional reliability dimension warrants consideration.

The authors noted the absence of intra-reader analyses and the restriction of the LLM evaluation to one model and a fixed prompt structure. These limitations concern human re-scoring and generalizability across readers, models, and prompts. A separate issue is LLM intra-model repeatability: run-to-run variability when the same article is scored repeatedly under an identical prompt and model configuration. The report does not state whether replicate scoring runs were performed or retained; consequently, the magnitude and source of any run-to-run variation cannot be determined [1]. Independent temporary sessions with memory disabled may reduce carry-over between articles, but they do not establish repeatability.

Earlier studies illustrate why these reliability dimensions should be evaluated separately. Mese and Kocak performed two independent ChatGPT-4o assessments of each article and directly evaluated intra-model agreement for RQS [2]. In another METRICS study, the same investigators compared ChatGPT-4 and NotebookLM with a consensus assessment by two radiologists [3]. The former design addresses within-model repeatability, whereas the latter evaluates cross-model differences and agreement with experts. These facets cannot be inferred from a single human-LLM agreement estimate.

The reported ICCs used a two-way random-effects, absolute-agreement, single-measurement specification [1]. This choice requires a clear definition of the intended rater population. Koo and Li provide a framework for selecting and reporting ICC forms [4]. In this setting, it is not self-evident that a fixed, named LLM and predefined expert groups should be regarded as random samples. Moreover, the Main Reader and Secondary Reader groups may provide a group-level expert reference rather than scores from individual raters. Clarifying whether the final expert scores were averaged, reached by consensus, or assembled from domain-specific item assessments is important. The ICCs therefore appear to compare an individual LLM output with a group-level expert reference rather than with an individual expert reader. This interpretation does not invalidate the analysis, but it narrows the inference for LLM-assisted appraisal. If individual reader-level item scores are available, reporting LLM agreement with each reader would help separate rubric-related from reader-related disagreement.

The reported item-level discrepancies make this distinction particularly important. ChatGPT tended to overestimate METRICS items involving subjective judgement while underestimating RQS items requiring clinical interpretation [1]. A single output may be pragmatic, but any variability associated with a single pass should be quantified before it is interpreted as reliable. Replicate assessments could distinguish stable rubric-related differences from stochastic output variation. Stable discrepancies may call for rubric clarification, prompt refinement, evidence-grounded scoring, or targeted expert adjudication. If outputs vary materially, repeated inference and aggregation may reduce random variation, but they cannot correct systematic item-level bias.

Future validation studies should preserve repeated LLM runs from the original evaluation period and document the model version, prompt, and relevant sampling settings. Where supported, a fixed random seed and a dated model snapshot should also be reported. Without this information, variation due to model updates or configuration changes may be conflated with stochastic run-to-run variability. Analyses could report total-score ICCs with confidence intervals and item-level agreement measures matched to each item scale. Confidence intervals should account for clustering by article. Agreement with the expert reference should be presented separately for individual LLM runs and an aggregated LLM score. Complete run-level outputs, item scores, and source-text excerpts supporting each decision would facilitate independent error analysis.

Garello et al show that agreement with expert assessment differs between METRICS and RQS and that LLM-generated scores require cautious interpretation. Distinguishing run-to-run repeatability from concordance with a group-level expert reference would provide a more complete evidential basis for evaluating scalable LLM-assisted radiomics appraisal. A brief author response clarifying whether repeated scoring runs were performed or retained could indicate whether a repeatability re-analysis is feasible.

## Data Availability

Data sharing does not apply to this article as no datasets were generated or analysed during the current study.
